# ﻿A new species of *Kodormus* Barber, with a redescription of the genus, taxonomic notes, and a key to the species of the genus (Hemiptera, Heteroptera, Reduviidae, Stenopodainae)

**DOI:** 10.3897/zookeys.1181.108463

**Published:** 2023-10-06

**Authors:** Hélcio R. Gil-Santana, Jean-Michel Bérenger, Jader Oliveira

**Affiliations:** 1 Laboratório de Diptera, Instituto Oswaldo Cruz, Av. Brasil, 4365, 21040-360, Rio de Janeiro, RJ, Brazil Laboratório de Diptera, Instituto Oswaldo Cruz Rio de Janeiro Brazil; 2 IRD, AP-HM, SSA, Vitrome, IHU Méditerranée Infection, Aix-Marseille Université, Marseille & Laboratoire d’Entomologie du Museum National d’Histoire Naturelle, Paris, France Aix-Marseille Université Marseille France; 3 Universidade de São Paulo, Faculdade de Saúde Pública, Laboratório de Entomologia em Saúde Pública, São Paulo, SP, Brazil Universidade de São Paulo Sao Paulo Brazil; 4 Laboratório de Parasitologia, Universidade Estadual Paulista “Julio de Mesquita Filho”, Faculdade de Ciências Farmacêuticas UNESP/FCFAR, Rodovia Araraquara Jaú, KM 1, 14801-902, Araraquara, SP, Brazil Universidade Estadual Paulista “Julio de Mesquita Filho” Araraquara Brazil

**Keywords:** Male genitalia, Neotropics, *Nitornus*, *Rhyparoclopius*, sexual dimorphism

## Abstract

*Kodormusdavidmartinsi***sp. nov.** is described. Taxonomic notes on the other species of *Kodormus* Barber, 1930, including the description of their male genitalia, are provided. The record of *Kodormusbruneosus* Barber, 1930 from Brazil and information about the female of the species are presented for the first time. A redescription of *Kodormus* and a key for its species are provided. Photographs of the holotypes of *K.barberi* (Costa Lima, 1941), *K.bruneosus*, and of a paratype of *K.oscurus* Maldonado & Bérenger, 1996 are presented.

## ﻿Introduction

Approximately 114 genera belonging to the assassin bug subfamily Stenopodainae (Hemiptera: Heteroptera: Reduviidae) have been described, with the majority of them inhabiting the tropics (Gil-Santana and Oliveira 2016; Schuh and Weirauch 2020). Their diversity is greatest in Africa and America (Giacchi 1987), particularly in the Amazon basin of South America (Bérenger 2001). Currently twenty genera are recognized as valid in the New World, which are separated in the key presented by Gil-Santana and Oliveira (2016). The taxonomy, general morphology, and the scarcely available biological data for American Stenopodainae were reviewed by Giacchi (1987).

Many Stenopodainae appear to be closely associated with the soil, often being covered with soil or sand or various types of debris. Most species are known from light trap collect, with males being captured much more commonly than females, and little is known regarding their biology (Giacchi 1987; Bérenger 2001; Schuh and Weirauch 2020). In *Kodormus* Barber, 1930, only females of *K.bruneosus* were collected (Barber 1930; this work). Giacchi (1987) stated that the sexual dimorphism is quite developed among species of Stenopodainae, including several characteristics, such as females being larger and more robust than males, while the antennal vestiture of males is more developed and differentiated.

*Kodormus* currently includes three species: *K.barberi* (Costa Lima, 1941), *K.bruneosus* Barber, 1930, and *K.oscurus* Maldonado & Bérenger, 1996 (Maldonado 1990; Bérenger and Maldonado 1996). In the present paper, *K.davidmartinsi* sp. nov. from Brazil is described based on two male specimens. Additionally, morphological remarks of the female of a species of *Kodormus* (*K.bruneosus*) are given for the first time. Taxonomical notes on *K.barberi*, *K.bruneosus*, and *K.oscurus*, including the description of their male genitalia are provided. *Kodormusbruneosus* is recorded from Brazil for the first time. A redescription of *Kodormus* and a key for its species are also furnished.

## ﻿Materials and methods

The male holotype of *Kodormusbruneosus* (Figs 51, 52) currently deposited in the National Museum of Natural History (**NMNH**), Smithsonian Institution, Washington, DC, USA, was directly examined and photographed by the third author. The holotype of *Kodormusbarberi* (Figs 1, 2), deposited in the “Coleção Costa Lima” in the “Coleção Entomológica do Instituto Oswaldo Cruz” (**CEIOC**), Rio de Janeiro, Brazil; holotype and paratype (Figs 91–104) of *K.davidmartinsi* sp. nov., and non-type specimens of *K.barberi* (Figs 3, 44–50) and *K.bruneosus* (Figs 53–62, 68, 73–85) were examined and their figures produced by the first author. Observations were made using a stereomicroscope (Zeiss Stemi) and a compound microscope (Leica CME). Measurements were made using a micrometer eyepiece. Photographs were taken using digital cameras (Nikon D5200 or D5600 with a Nikon Macro Lens 105). Dissections of the male genitalia were made by first removing the pygophore from the abdomen with a pair of forceps and then clearing it in 20% NaOH solution for 24 h. The dissected structures were studied and photographed in glycerol and photographed using a digital camera (Sony DSC-W830). Drawings were made using a camera lucida. Images were edited using Adobe Photoshop CS6.

Photographs and scanning electron microscopy (SEM) images of a non-type specimen of *K.barberi* (Figs 5–43) and tibial pads of *K.bruneosus* (Figs 66, 67, 70) were obtained by the third author. The photographs were taken using a stereomicroscope (Leica 205A) with a digital camera. The SEM images were obtained by cleaning the specimen in an ultrasound machine. Subsequently, the samples were dehydrated in alcohol, dried in an incubator at 45 °C for 20 min, and fixed in small aluminum cylinders with transparent glaze. Sputtering metallization was then performed on the samples for 2 min at 10 mA in an Edwards sputter coater. After this process, the samples were studied and photographed using a high-resolution field emission gun scanning electron microscope (SEM; JEOL, JSM-6610LV), similarly as described by Rosa et al. (2010, 2014).

Non-type specimens of *K.bruneosus* and two male paratypes of *K.oscurus* were examined and imaged by the second author. Photographs of their habitus (Figs 86, 87, 105) were taken with a Canon 5D mark II with a Canon macro lens 100; the image comparing pedicels of a female and a male of *K.bruneosus* (Fig. 88) was taken with a Canon D40 with a Canon macrolens MP-E 65; photos were stacked using combineZ program. Photographs of the genital structure of a female of *K.bruneosus* (Figs 89, 90) and of a male of *K.oscurus* (Fig. 108) were taken with a Canon 5D mark II with a Laowa 25 mm f2.8 ultra macro lens, while the images of the phallus and paramere (Figs 106, 107, 109, 110) of the male of *K.oscurus* with Zeiss Axio Zoom V16 equipment. The SEM images of some structures of *K.bruneosus* (Figs 63–65, 69, 71, 72) were taken with TM 4000 Plus Hitachi tabletop microscope.

A photograph of a living specimen of *K.barberi* was taken by Dr. Ricardo Brugnera (Insetos do Brasil Project) (Fig. 4), which can be freely accessed with information about the data where it was found at https://www.inaturalist.org/observations/107772379.

The type specimens of *Kodormusdavidmartinsi* sp. nov. will be deposited as follows: male holotype in the Collection of National Museum of the Federal University of Rio de Janeiro, Rio de Janeiro, Brazil (**MNRJ**) and 1 male paratype in the “Coleção de Triatomíneos do Instituto Oswaldo Cruz”, Rio de Janeiro, Brazil (**CTIOC**) of the “Laboratório Nacional e Internacional de Referência em Taxonomia de Triatomíneos” (**LNIRTT**) at Oswaldo Cruz Institute, Rio de Janeiro, Brazil.

The general morphological terminology used mainly follows Giacchi (1987) and Schuh and Weirauch (2020). The [visible] segments of the labium are numbered as II to IV, given that the first segment is lost or fused to the head capsule in Reduviidae (Weirauch 2008; Schuh et al. 2009). The terminology applied to the male genital characteristics mainly follows Lent and Wygodzinsky (1979), Gil-Santana (2012), and Gil-Santana and Oliveira (2016).

Additional acronyms of the depository collections, not mentioned above, are the following:

**J-MB** Jean-Michel Bérenger private collection, France;

**MNHN**Museum national d’Histoire naturelle, Paris, France;

**RBINS**Institut Royal des Sciences Naturelles de Belgique, Bruxelles, Belgium;

**SEAG** Société Entomologique Antilles-Guyane, Guyane.

When describing label data, a slash (/) separates the lines and a double slash (//) different labels, and comments or translations to English of the label data are provided in square brackets ([]).

## ﻿Taxonomic account

### ﻿ Kodormus

Taxon classificationAnimaliaHemipteraReduviidae

Barber, 1930

9B4894F0-32D7-5689-BE30-892A8F30A88B

Kodormus Barber, 1930: 151 [key], 213–214 [description]; Costa Lima 1940: 166, footnote [Kodormus considered identical to Otiodactylus Pinto, 1927]; Costa Lima 1941: 337–338 [Kodormus recognized as diverse from Otiodactylus but alleged as possessing the same characteristics of Ocrioessa Bergroth, 1918]; Costa Lima and Campos Seabra 1944: 507 [Kodormus recognized as distinct from Ocrioessa], 510 [key]; Costa Lima and Campos Seabra 1945: 159 [checklist]; Wygodzinsky 1949: 66 [catalog]; Putshkov and Putshkov 1985: 104 [catalog]; Giacchi 1985: 68–69 [redescription]; Maldonado 1990: 506 [catalog]; Wygodzinsky and Giacchi 1994: 7 [key], 8 [checklist]; Bérenger and Maldonado 1996: 37 [comparison with other genera]; Froeschner 1999: 227 [catalog]; Forero 2004: 166 [diagnosis], 167 [new record from Colombia], 191 [key]; Gil-Santana et al. 2015: 337 [citation], 341 [key]; Gil-Santana and Oliveira 2016: 501, 502 [citations], 505 [key].

#### Type species.

*Kodormusbruneosus* Barber, 1930: 214–216, by original designation.

#### Diagnosis.

This genus can be separated from other genera of the New World by the following set of characters. Body somewhat elongated, ~ 2–3× as long as maximum width, slightly flattened dorsoventrally. Head large, anteocular portion longer than postocular; antennal scape shorter than anteocular portion; eyes prominent, shortly setose; labial segment II [first visible] shorter than the others combined; postocular region broad; ramose setigerous processes posterolaterally behind eyes; anterior lobe of pronotum with anterior angles prominent, anterior and lateral margins covered with a row of setigerous tubercules, and a pair of tubercles on its disc; pronotum wider across humeri than along midline; humeral angles protruding; prosternum behind coxae shorter than length of coxae; evaporatory area of metapleuron large, sooty black; fore femora strongly incrassate, robust, at least twice as thick as middle and hind femora; fore and hind tibiae curved, small tibial pads on apices of fore and middle tibia. Abdomen broad, with a more or less expanded connexival margins, which are denticulate and/or lobulated at posterolateral angles of segments II–VI; in male, posterior margin of the abdominal segment VII almost or completely covering the pygophore in dorsal view and with a slightly bilobate shape; in the (known) females, the genital area is visible from above and conical.

#### Redescription.

Body somewhat elongated, ~ 2–3× as long as maximum width, slightly flattened dorsoventrally. General color pale to dark brownish with darkened and pale portions; a clear, generally whitish rounded or subrounded spot above the approximately mid-portion of the outer cell of the membrane of hemelytra. Integument dull, body and legs, except tarsi, generally covered with short, rounded tubercles, each with a short pale apical scale-like seta (setigerous tubercles), scale-like setae, and on some areas, simple setae too. Some glabrous areas, such as the interocular sulcus or forming lines on head, thoracic sterna and femora, subrounded to irregular areas on anterior lobe of pronotum, pleura and abdomen. The integument is generally rugous where there are setigerous tubercles and smooth in the glabrous portions. Simple erect or curved setae are present on labium, antennal segments II–IV, fore tibiae and tarsi. ***Head*** subcylindrical; a little longer than wide; shorter than pronotum; anteocular region ~ 2× longer than postocular region, the latter wider than the former. Mandibular plates (jugae sensu Barber 1930; Costa Lima 1941; tylus sensu Giacchi 1985) prominent, divergent, tapering. A small lateral protuberance on antenniferous with setigerous seta(e). Antenna inserted far from eye, somewhat anterior to middle point of anteocular portion, laterally; scape thickened at distal 2/3, somewhat curved at middle 1/3, shorter than anteocular region and covered with pale scale-like setae; pedicel, longer than other segments, > 2× longer and slenderer than the scape, straight at basal 1/2, somewhat curved at middle portion or distal 1/2 and slightly thickened apically; in male, with very numerous, pale to whitish, thin, long, erect to somewhat curved setae, forming a dense pubescence covering almost all the segment, except on anterodorsal surface, where these setae are scarcer and there are 2–4 irregular rows of sparse stout darkened and stiff long setae, in which one or two rows are composed by setae serrate at their distal portion, while the setae of the other rows are uniform; at distal portion of the pedicel the stout setae are less numerous and serrate setae are absent; apex covered by shorter curved pale setae. In the female, the pedicel has sparse scale-like setae and, at the distal portion, scattered straight or somewhat curved short pale setae. Flagellomeres much thinner, cylindrical, straight, subequal in length, each a little shorter than the scape, with few scattered long, erect, stouter setae and a pubescence of simple setae which is formed by thin, short to moderately longer setae on basiflagellomere and generally shorter and even thinner setae on distiflagellomere; the apex of the latter acutely pointed. Clypeus depressed, with a pair of more developed setigerous tubercles. Eyes globose, rounded in dorsal view; suboval in lateral view, extending somewhat on the lower surface of the head, with sparse scale-like setae among facets. Transverse sulcus not very deep, somewhat curved; more sinuous at lateral portions, reaching eyes at their inner posterior angle. Ocelli moderately large, prominently elevated, each ocellus separate from the other for a distance wider than the width of each of them. Labial segment II [first visible] thicker and shorter than the others combined, reaching approximately level of anterior portion of eye; its length subequal to that of the segment III; the latter thinner toward apex; segment IV slender, ~ 1/2 as long as segment III, tapering; its apex reaching stridulatory sulcus on approximately its middle 1/3. Postocular region of the head converging behind eyes to neck, rounded on dorsal view, with one or two conspicuous ramose setigerous processes posterolaterally at each side; above the latter, between eyes and posterior margin, a serial line of somewhat more developed setigerous tubercles. On ventral surface of head, 4–16 large conspicuous setigerous tubercles, generally grouped by transverse pairs, but sometimes, besides some pairs, an isolated tubercle is present at only one side. Two or more of these tubercles generally lie anteriorly to the eyes, and the more posterior pair lies between eyes, near their posterior margin. While in the most posterior pair, the tubercles are very close to each other or even contiguous; in the other pairs, they are clearly separated from each other. ***Thorax***: pronotum wider than long, with anterolateral angles prominent; a pair of tubercles on disc of fore lobe; pronotum wider across humeri than along midline; humeral angles pointed or more prominent. Anterior collar with a variable number of somewhat more developed setigerous tubercles, which also form single rows on the lateral margins of fore lobe of pronotum and on the antero-lateral margin of propleura. Transverse furrow between fore and hind lobes of pronotum shallow, interrupted laterally by a pair of faint submedian ridges; the latter run on approximately the proximal 1/3 of the hind lobe. Fore lobe with a median very thin and somewhat deep midlongitudinal sulcus on the approximately distal 1/2 of fore lobe; sinuate linear ridges covered with setigerous tubercles, narrow and glabrous areas among them and between the most external ridges; lateral margin covered by a row of setigerous tubercles. Supracoxal lobes not prominent; anterior portion of fore supracoxal lobe with a group of conspicuous tubercles, similar to the ramose setigerous of the head. Scutellum subtriangular, longer than wide, with an erect apical tubercle; metascutum also with a short tubercle. Propleura moderately declivous, reaching ventral side laterally and posteriorly to fore coxae. Meso- and metapleura almost vertical; evaporatory area of metapleura large, sooty black. Anterior prosternal processes moderately elongated and curved downwards at apex. Prosternum behind coxae shorter than length of coxae. Stridulitrum long. Mesosternum flat; metasternum slightly prominent at median portion. ***Legs***: fore coxae close, separated from each other by the prosternum, which surpasses fore coxae, by a short distance; middle coxae inserted somewhat less close to each other than the fore coxae; hind coxae inserted very distant from each other. Coxae with two or three ill-defined glabrous longitudinal lines; large setigerous tubercles on fore coxae, more numerous or only present anteriorly. Trochanters with glabrous areas; fore trochanters with two pairs of spiny tubercles on internal surface. Fore femora fusiform, strongly incrassate, at ≥ 2× thicker than middle and hind femora; ventrally with spiny, relatively small, rounded tubercles, including a basal group of 3–5 elements, a midline row with 5–8 elements and some others close to this row on anterior surface; at apex, a lateroventral pair of conspicuous setigerous processes. Fore and middle femora approximately as long as respective tibiae. Scale-like setae on femora and middle and hind tibiae very numerous and generally longer. Fore tibiae with smaller, less numerous or without tubercles and generally less setose than other tibiae. Tarsi with scattered scale-like setae dorsally and stout, straight or slightly curved setae, more numerous, sometimes forming tufts, ventrally. All femora with glabrous lines, which are larger and more evident on fore femora and straight, thinner, and less evident or partly interrupted on middle and hind femora. On fore femora, a ventral and two dorsal of these glabrous, somewhat shiny, lines are generally present. Mid and hind femora slender, straight, somewhat thickened subdistally, generally with some more developed subapical setigerous processes, ventrally. Fore and hind tibiae curved, middle tibiae slightly curved or sometimes straight; all of them compressed dorsoventrally, except at base, generally with a median shallow narrow longitudinal furrow on each lateral surface, except at base, with small tibial pads on fore and middle legs. Fore tarsi two- or three-segmented; middle and hind tarsi three-segmented. Ratio of tarsal segments (approximately): fore tarsi: 1:3 (when two-segmented) / 1:1.5:2.8 (when three-segmented); middle tarsi: 1:1.4–1.6:1.9–2.0; hind tarsi: 1:1.2–1.5:1.8–2.2. Hemelytra with discal cell closed, although the distal cross vein may be indistinct and the cell seems open; corium generally with sparse small scale-like setae, which are more numerous on lateral portion; membrane glabrous. ***Abdomen***: suboval in shape, flattened; segments gradually widening to apex of segment V, then strongly shortening in the next two segments, towards apex; first tergite narrow, integument with shallow longitudinal ridges; tergites II–VI glabrous at median portion in variable extent; scars of dorsal abdominal gland openings on median anterior margins of tergites IV and V very small; connexival margins prominently denticulate and/or lobulated at posterolateral angles of segments II–VI; progressively larger from segment II to V, the latter, although variably in shape among the species, is always the largest, while that on segment VI has a dimension similar or slightly larger in comparison to the prominences on segments II and III. Sternite II (first visible) < 1/3 as long as the sternite III. Sternites II–VI with a median longitudinal narrow pronounced keel; spiracles on sternites II–VII elliptical, prominent, diagonally oriented in relation to the abdominal margin, approximately at medial point between the intersegmental furrows; their margins darkened, even in individuals in which the surrounding integument is pale. In male, posterior border of segment VII straight or curved at median portion, latero-distal margins curved or acute; eighth sternite slightly sinuous on median portion of posterior margin. In the (known) females, the genital area is visible from above and conical. ***Male genitalia***: Genital capsule only visible in ventral view and when in situ, with parameres visible in posterior view; exposed portion of pygophore sub-rounded, covered with setigerous tubercles with scale-like setae; in dorsal view, between anterior and genital openings, a moderately narrow bridge; laterodorsal margin of pygophore, between the bridge and the insertion of parameres, with numerous variably long erect simple setae. Proctiger subsquared with several long setae on approximately its distal 1/3. Medial process of pygophore only visible via dorsal view, with adjacent sparse erect setae, directed upwards, situated just below the paramere apices, subtriangular, triangular or spiniform in anterior view; straight, elongated, thin, and with apex acute in lateral view. Paramere apices close in resting position; in ventral view only the posterior margins of their apices are visible. Parameres symmetrical, very curved in median portion, with a sclerotized moderately large subapical blunt prominence on internal surface; glabrous on approximately basal 1/3 and generally covered with scale-like setae on the exposed surface and scattered, straight, moderately short to longer, simple, erect, thin setae, which are somewhat more numerous around the subapical prominence. Phallus: articulatory apparatus short, with a short basal plate bridge and somewhat longer basal plate arms; pedicel longer than articulatory apparatus, slightly enlarged towards apex, with deep transverse ridges, curved in lateral view and subrectangular in dorsal and ventral views. Gonopore process slightly sclerotized, broad. Dorsal phallothecal sclerite subrectangular, moderately sclerotized. Struts as a pair of elongated arms, fused distally; subcylindrical in approximately basal 2/3 and somewhat enlarged towards apices, which are rounded. Endosoma formed only by its wall, which is smooth and very wrinkled, distal margin more coarsely rugous and sclerotized, shortly prolonged ventrally by an almost imperceptible fold which leans against the main portion of the endosoma.

### ﻿Key to the species of *Kodormus*

**Table:** 

1	A single ramose setigerous process posterolaterally behind each eye (Figs 14, 94); fore tarsus with two segments only (Figs 32, 33, 95); connexivum of segment V lobulated at external margin (Figs 1, 3, 4, 39, 91–93, 96, 97)	**2**
–	A pair of ramose setigerous processes posterolaterally behind each eye; fore tarsus three-segmented (Fig. 68); connexivum of segment V acutely prominent at external margin (Figs 51, 53–55, 73, 74, 86, 105)	**3**
2	Integument generally covered by more developed and larger tubercles, including dorsal surface of head and legs (Figs 5, 6, 11–14, 17, 26, 28, 29); processes on disc of fore lobe of pronotum, humeral angles and apex of scutellum long and conspicuous (Figs 17–25); latero-distal margins of abdominal segment VII acute (Figs 40, 41) ; medial process of pygophore spiniform in anterior view (Fig. 43)	***K.barberi* (Costa Lima, 1941)**
–	Integument with scattered small tubercles; processes on disc of fore lobe, humeral angles and apex of scutellum short (Fig. 92); latero-distal margins of abdominal segment VII rounded (Fig. 96); medial process of pygophore subtriangular in anterior view (Fig. 100)	***K.davidmartinsi* sp. nov.**
3	General coloration brownish; latero-distal angles of connexivum less prominent (Figs 51, 53–55)	***K.bruneosus* Barber, 1930**
–	General coloration much darker than in preceding species; latero-distal angles of connexivum more prominent (Fig. 105)	***K.oscurus* Maldonado & Bérenger, 1996**

### ﻿ Kodormus barberi

Taxon classificationAnimaliaHemipteraReduviidae

(Costa Lima, 1941)

53AC19B3-9515-5BF0-AAB5-36044986A156

Figs 1–4 , 5–10 , 11–16 , 17–26 , 27–33 , 34–41 , 42–50

Ocrioessa barberi Costa Lima, 1941: 339–341, figs 2, 5–6; Rodrigues et al. 2017: 188 [catalog of type specimens; present combination cited], fig. 75 [holotype, dorsal view], table 1 [citation; present combination cited].
Kodormus barberi ; Costa Lima and Campos Seabra 1944: 507 [new combination]; Costa Lima and Campos Seabra 1945: 159 [checklist; new combination reinforced]; Wygodzinsky 1949: 66 [catalog]; Maldonado 1990: 506 [catalog]; Bérenger and Maldonado 1996: 35 [citation], figs 9, 37 [distinguishing features]; Gil-Santana and Alencar 2001: 173 [checklist; as a misidentification of K.davidmartinsi sp. nov.; see below]; Forero 2004: 166 [citation].

#### Notes.

**Figures 1–4.**
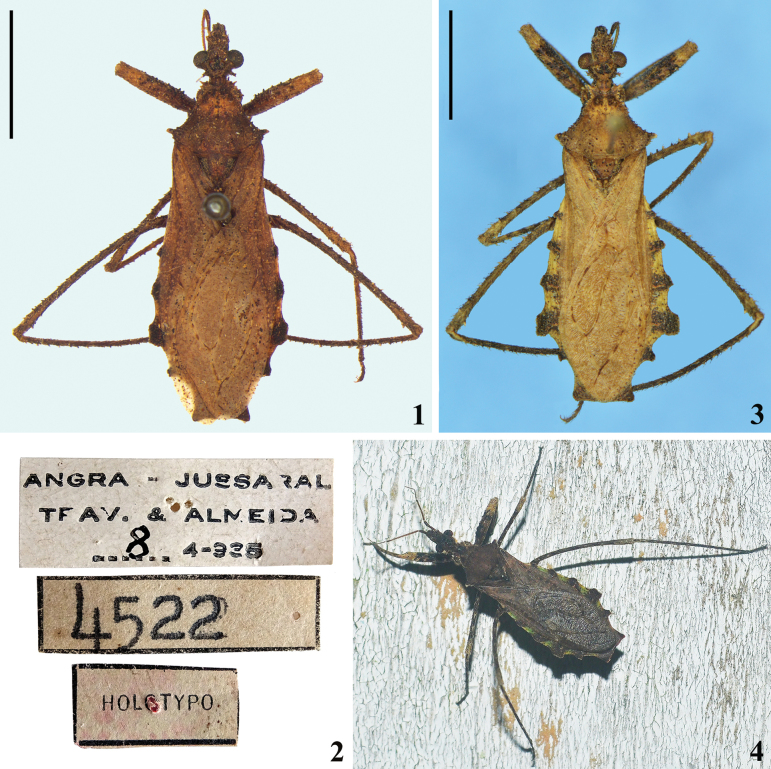
*Kodormusbarberi* (Costa Lima, 1941) **1, 2** male holotype deposited in CEIOC**1** dorsal view **2** labels **3, 4** non-type specimen, males, dorsal view **4** living specimen. Scale bars: 5.0 mm (**1, 3**).

Costa Lima (1941) described *Ocrioessabarberi* based on a male holotype (Figs 1, 2) and a male paratype, both from southeast Brazil (States of Rio de Janeiro and São Paulo, respectively). It is noteworthy that Costa Lima (1941) argued that *Kodormusbruneosus* should belong to *Ocrioessa*, while *O.barberi* would be extremely close to this species. He additionally stated that *O.barberi* could not be subsumed to the other two species of *Ocrioessa* because both presented pads in fore tibiae (Barber 1930) while the latter were absent in fore tibiae of *Kodormus*, accordingly with its description (Barber 1930). Despite these statements, Costa Lima (1941) did not propose any formal synonym between *Ocrioessa* and *Kodormus*. Costa Lima and Campos Seabra (1944), however, concluded that *Kodormus* was really distinct from *Ocrioessa*, establishing the new combination, *Kodormusbarberi*, which was reinforced by Costa Lima and Campos Seabra (1945).

#### Type material examined.

*Ocrioessabarberi* Costa Lima, 1941. Brazil: ***Male holotype***: [printed label] ANGRA – JUSSARAL / TRAV. [= Travassos] & ALMEIDA [leg.] / 8 [handwritten] 4 -[1]935 // [framed typewritten label] 4522 // [framed printed label] HOLOTYPO (CEIOC).

#### Additional specimens.

*Kodormusbarberi* (Costa Lima, 1941). Brazil: Rio de Janeiro: *Kodormus* / *barberi* / (Costa Lima) [handwritten] / Wygodzinsky det. [printed] ’64 [handwritten] // J. F. Zikán [printed vertically at left side] / Itatiaya [printed] 700 m [handwritten] / [printed] E. [State of] Rio [de Janeiro] – Brasil [Brazil] / [handwritten] 15.-X.-1935 Z. [?] // [framed printed label] CTIOC / N°. 855, 1 male; *Kodormus* / *barberi* / (Costa Lima) [handwritten] / Wygodzinsky det. [printed] ’64 [handwritten] // J. F. Zikán [printed vertically at left side] / Itatiaya [printed] 700 m [handwritten] / [printed] E. [State of] Rio [de Janeiro] – Brasil [Brazil] / [handwritten] 13.-IX.-1941 Z. [?] // [printed label] Coleção [Collection] J. F. Zikan // [framed printed label] CTIOC / N°. 856, 1 male; São Paulo: [printed label] SALESÓPOLIS (BORACÉA) / S. PAULO – 24–IX–[1]946 / TRAVASSOS & VENTEL [leg.] // [framed printed label] CTIOC / N°. 851, 1 male; *Kodormus* / *barberi* / (Costa Lima) [handwritten] / Wygodzinsky det. [printed] ’64 [handwritten] // [printed label] SALESÓPOLIS (BORACÉA) / S. PAULO – 24–9–[1]946 / TRAVASSOS &VANSOLINI [*sic*], [leg.] // [printed label] Instituto Osvaldo Cruz // [handwritten label] desenhado [drawn] // [framed printed label] CTIOC / N°. 852, 1 male; [printed label] SALESÓPOLIS (BORACÉA) / S. PAULO – 24–9–[1]946 / TRAVASSOS &VANSOLINI [*sic*], [leg.] // [framed printed label] CTIOC / N°. 853, 1 male; *Kodormus* / *barberi* / (Costa Lima) [handwritten] / Wygodzinsky det. [printed] ’64 [handwritten] // [printed label] SALESÓPOLIS (BORACÉA) / S. PAULO – 24–9–[1]946 / TRAVASSOS &VANSOLINI [*sic*], [leg.] // [framed printed label] CTIOC / N°. 854, 1 male (CTIOC).

#### Diagnosis.

*Kodormusbarberi* can be separated from other species of the genus by the more developed and larger integumental setigerous tubercles, longer and more conspicuous processes on the disc of fore lobe of pronotum, humeral angles, scutellum and acute latero-distal margins of abdominal segment VII.

#### Description.

**Figures 5–10.**
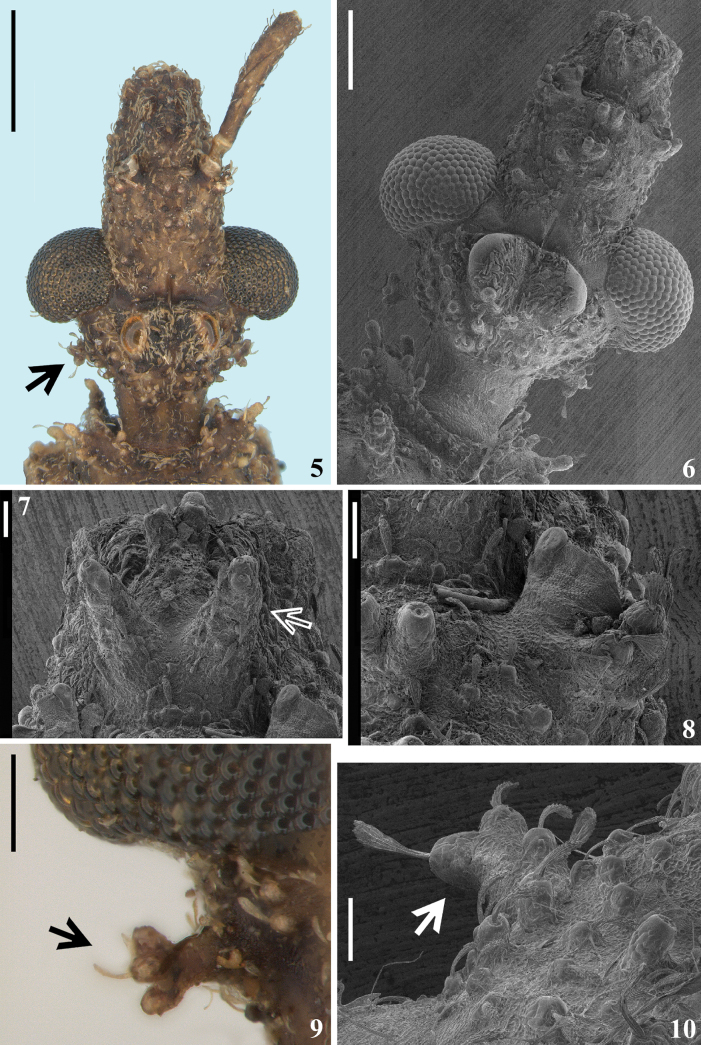
*Kodormusbarberi*, male, head **5–8** dorsal view **5** antennae, except right scape, excluded, the arrow indicates a posterolateral ramose setigerous process **6–8** antennae excluded **7, 8** anteocular portion **7** the arrow points to a mandibular plate **8** antenniferous **9, 10** posterolateral ramose setigerous process on postocular portion, pointed by an arrow **9** ventral view **10** dorsoposterior view. Scale bars: 2.0 mm (**5**); 0.5 mm (**6, 9**); 0.1 mm (**7, 8, 10**).

**Figures 11–16.**
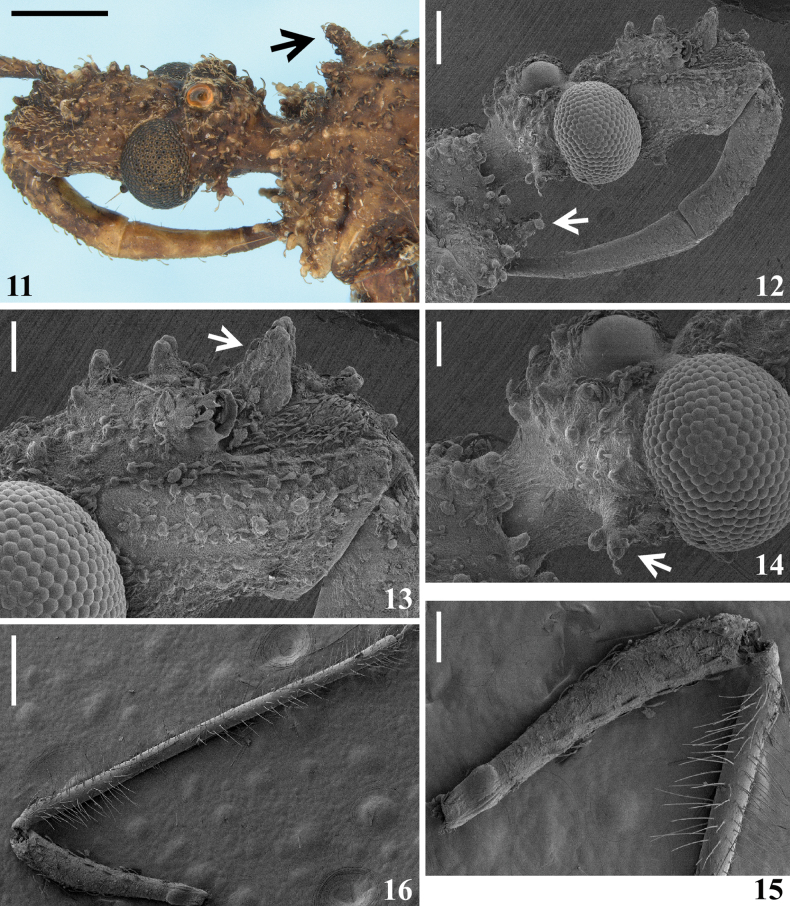
*Kodormusbarberi*, male **11, 12** head and anterior portion of pronotum **11** laterodorsal view, the arrow indicates a tubercle of fore lobe of pronotum **12** lateral view, the arrow indicates an anterior prosternal process **13, 14** portions of head **13** anteocular portion, lateral view, the arrow indicates a mandibular plate **14** postocular portion, dorsolateral view, the arrow indicates a posterolateral ramose setigerous process **15, 16** antennal segments, lateral view **15** scape and basal portion of pedicel **16** scape and pedicel. Scale bars: 2.0 mm (**11**); 0.5 mm (**12, 16**); 0.2 mm (**13–15**).

**Figures 17–26.**
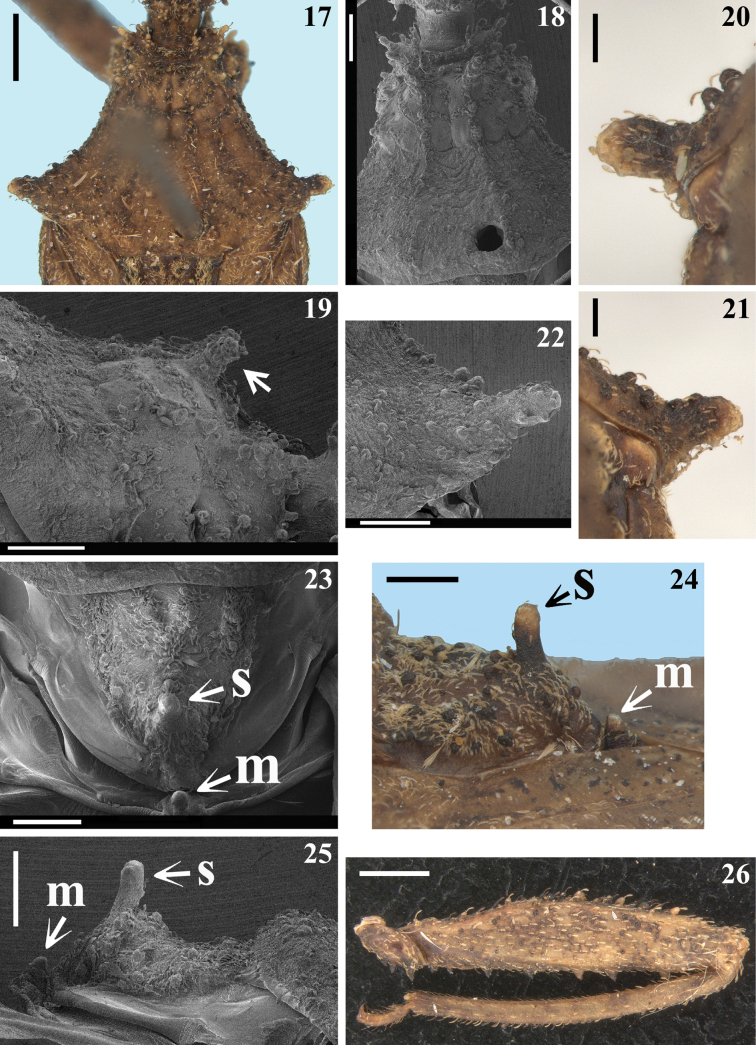
*Kodormusbarberi*, male **17–19** pronotum **17, 18** dorsal view **18** except humeral angles **19** central portion, lateral view, the arrow points to a tubercle of fore lobe **20–22** humeral prominences **20, 21** ventral view **22** dorsal view **23–25** scutellum and tubercle of metascutum **23** dorsal view **24, 25** lateral view **26** fore leg, lateral view. Abbreviations: **s**: apical tubercle of scutellum; **m**: tubercle of metascutum. Scale bars: 2.0 mm (**17, 26**); 1.0 mm (**24**); 0.5 mm (**18–23, 25**).

**Figures 27–33.**
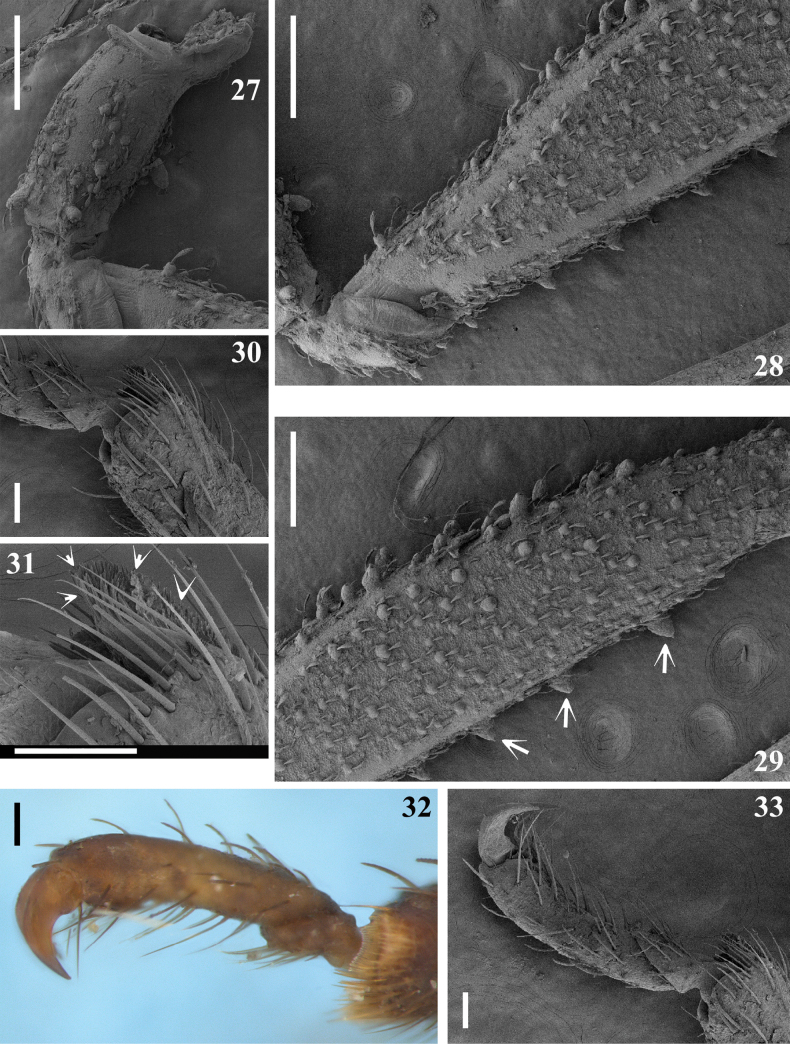
*Kodormusbarberi*, male, fore leg portions, lateral view **27** coxa and basal portion of trochanter **28** trochanter and basal portion of femur **29** middle and distal portion of femur, arrows point to ventral spiny rounded tubercles **30** apex of tibia and basal portion of tarsus **31** apex of tibia, the arrows point to the apical pad **32, 33** tarsus. Scale bars: 0.5 mm (**27–29**); 0.2 mm (**32**); 0.1 mm (**30, 31, 33**).

**Figures 34–41.**
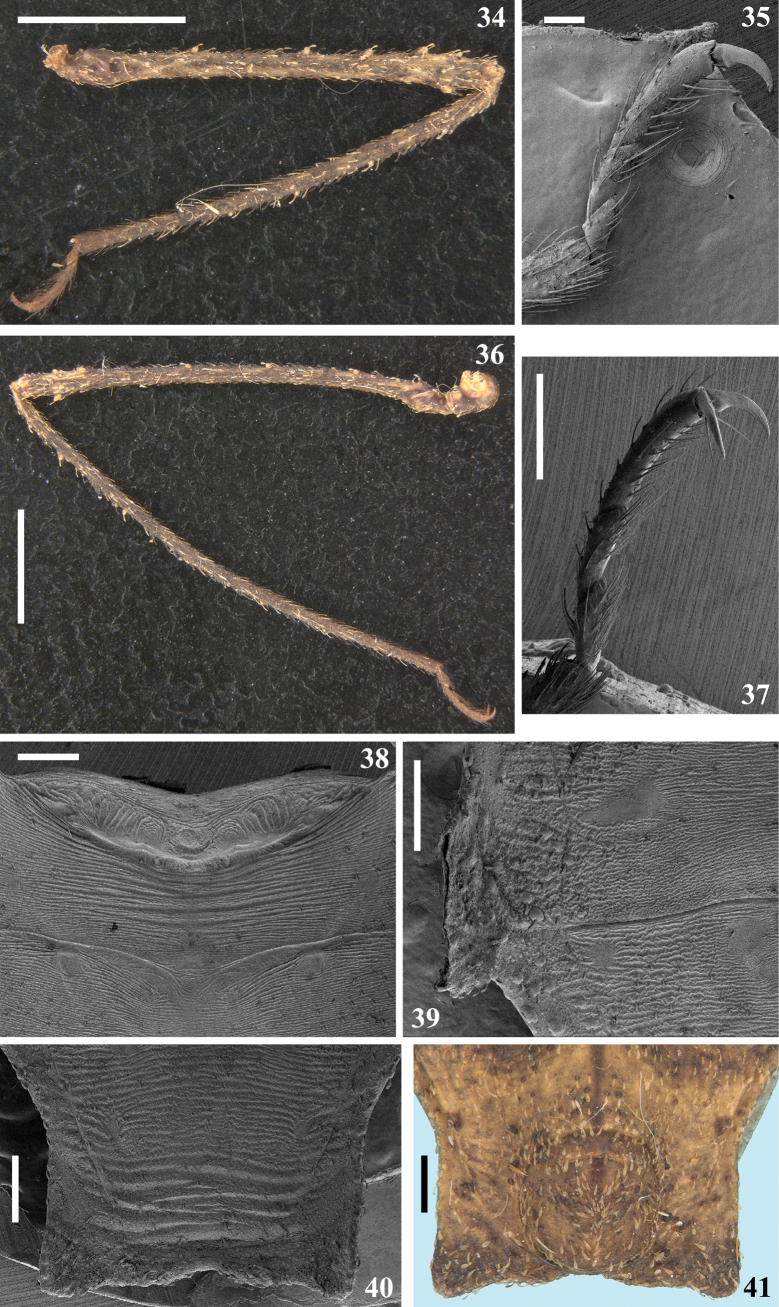
*Kodormusbarberi*, male **34–36** lateral view **34** middle leg **35** middle tarsus **36** hind leg **37** hind tarsus, lateroventral view **38–41** abdomen portions **38–40** dorsal view **38** tergite I, median portions of tergite II and basal half of tergite III **39** process of the connexival segment V and lateral portion of tergites V and VI **40, 41** apex of abdomen **41** ventral view. Scale bars: 5.0 mm (**34, 36**); 1.0 mm (**41**); 0.5 mm (**37–40**); 0.2 mm (**35**).

**Figures 42–50.**
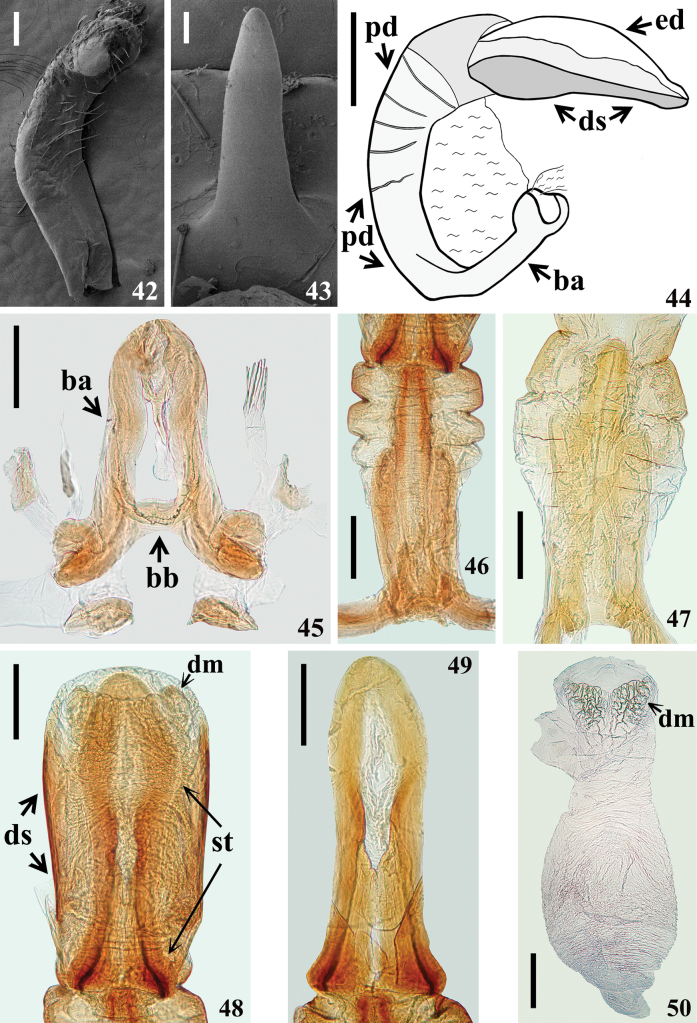
*Kodormusbarberi*, male genitalia **42** paramere, inner view **43** medial process of pygophore, anterior view **44** phallus, lateral view **45, 46** dorsal view **45** articulatory apparatus **46, 47** pedicel **47** ventral view **48** dorsal phallothecal sclerite, struts and endosoma **49** struts **50** endosoma. Abbreviations: **ba**: basal plate arm; **bb**: basal plate bridge; **dm**: distal margin of endosoma; **ds**: dorsal phallothecal sclerite; **ed**: endosoma; **pd**: pedicel; **st**: struts. Scale bars: 0.3 mm (**44**); 0.2 mm (**45–50**); 0.1 mm (**42**); 0.03 mm (**43**).

**Male.** Figs 1, 3–50. ***Total length***: 17.0–19.0 mm; maximum width of abdomen (between apices of connexival prominences of segment V): 5.5–6.8 mm. ***Coloration*** (Figs 1, 3–5, 11, 17, 20, 21, 24, 26, 34, 36, 41): generally brownish; scattered ill-defined and variable darkened and pale markings or portions along the body and legs; pedicel variably paler with apex darkened; apices of femora pale, more extensively on fore femora; apices of prominences of humeri, scutellum and metascutum paler; connexivum paler with prominences darkened; pale portions on fore femora and connexivum sometimes with a greenish to a yellowish tinge. ***Structure*** and ***vestiture***: Dorsal surface of head with several large setigerous tubercles (Figs 5, 6, 11–14). Postocular region of the head with only one posterolateral ramose setigerous process at each side (Figs 5, 6, 9, 10, 14). Tubercles on disc of fore lobe, elevated, thick and spiniform (Fig. 11). Humeral angle with an elongated and thick process (Figs 17, 20–22). Process of scutellum moderately elongated (23–25). Coxae, femora and tibiae (except fore tibiae) generally covered by numerous large setigerous tubercles (Figs 26–29, 34, 36). Middle tibiae slightly curved; straight in some individuals (Figs 1, 3, 34). Fore tarsi bi-segmented; the second segment ~ 3× as long as the first segment (Figs 32, 33). Hemelytra with distal cross vein variably distinct or not distinct; membrane of hemelytra varying from not reaching to slightly surpassing apex of abdomen (Figs 1, 3, 4). Connexival margins prominently lobulated at posterolateral angles of segments II–VI; short, but progressively larger from segment II to V, the latter, although variably in shape among the specimens, is always the largest, while that on segment VI has a dimension similar or slightly larger in comparison to the prominences on segments II–III (Figs 1, 3, 4, 39). Lateroapical margins of last abdominal segment prominent, acute or faintly curved (Figs 1, 3, 4, 40, 41). ***Male genitalia*** (Figs 41–50): medial process of pygophore small, straight, spiniform in anterior view (Fig. 43).

#### Comments.

In the original description of *K.barberi*, Costa Lima (1941) recorded the pads on fore tibiae as being absent. However, the examination of the holotype as well as additional non-type specimens allowed confirmation that a small pad is present on the fore and middle tibiae in this species (Fig. 31), while the fore tarsi are revealed to be bi-segmented (Figs 32, 33).

#### Distribution.

Brazil (States of Rio Grande do Sul, Rio de Janeiro and São Paulo) (Costa Lima 1941; this work; Insetos do Brasil Project).

### ﻿ Kodormus bruneosus

Taxon classificationAnimaliaHemipteraReduviidae

Barber, 1930

9B1DD0BC-63FB-5AC5-AA45-135A72E6A414

Figs 51–54 , 55–62 , 63–68 , 69–74 , 75–85 , 86–90

Kodormus bruneosus Barber, 1930: 214–216 [description]; Costa Lima 1940: 166, footnote [Kodormusbruneosus considered as being possibly identical to Otiodactylussignatus Pinto, 1927]; Costa Lima 1941: 337–338 [K.bruneosus very different from Otiodactylussignatus; should be included in Ocrioessa]; Wygodzinsky 1949: 66 [catalog]; Villiers 1971: 684 [misspelled as “*brunneosus*”; recorded from French Guiana]; Giacchi 1985: 69 [redescription of the male]; Maldonado 1990: 506 [catalog]; Bérenger and Maldonado 1996: 35 [citation], fig. 8, 37 [distinguishing features]; Froeschner 1999: 227 [catalog]; Forero 2004: 166–167, fig. 5.108 [citation, new record from Colombia]; Forero 2006: 36, fig. 58 [new record from Colombia]; Gil-Santana and Husemann 2023: 407, fig. 26 [new records from Ecuador and Peru].

#### Notes.

**Figures 51–54.**
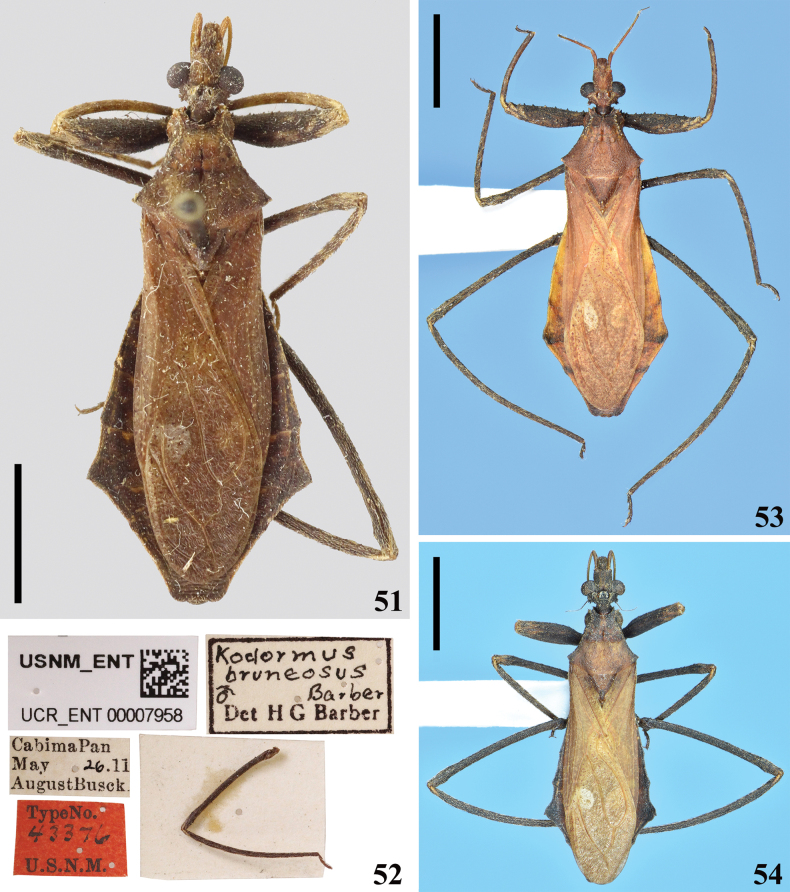
*Kodormusbruneosus* Barber, 1930 **51, 52** male holotype deposited in NMNH **51** dorsal view **52** labels and left hind leg glued to a card pinned with the specimen **53, 54** non-type specimens, males, dorsal view **53** specimen from Ecuador **54** specimen from Brazil. Scale bars: 5.0 mm (**51, 53, 54**).

*Kodormusbruneosus* was described based on three specimens: a male “Type” (Figs 51, 52), and as “Paratype”, a female and an additional male (Barber 1930). The use of the term “Paratype” in the singular must have been a typo. He probably meant to state both specimens as paratypes as he did in several other species described in the same paper. On the other hand, the male designated by him as the “Type” is regarded here as a holotype, following the Art. 73.1.1 of the International Code of Zoological Nomenclature (ICZN 1999), which defines that if an author states in the original publication that one specimen and only one is “the type” or uses some equivalent expression, that specimen is the holotype fixed by original designation.

#### Type material examined.

*Kodormusbruneosus* Barber, 1930. ***Male holotype***: Panama: [printed label] USNM_ENT, QR CODE / UCR_ENT 00007958 // [framed label] [handwritten] *Kodormus* / *bruneosus* / ♂ Barber / [printed] Det H G Barber // [almost completely printed label] CabimaPan [Cabima, Panama] / May 26 [handwritten].11 / AugustBusck // [red label] [almost completely printed label] TypeNo. / 43376 [handwritten] / U.S.N.M. (NMNH).

#### Additional specimens.

Brazil: Maranhão: Balsas, 08°48'41"S, 46°21'49"W, x.1996, leg. M. Eklein, 1 male; Feira Nova do Maranhão, Retiro, 07°00'31"S, 46°26'41"W, 29–30.xi.1995, leg. M. Eklein, 1 male; Mato Grosso, Diamantino, Alto Rio Arinos, 14°25'S, 56°29'W, 30.iv.2002, E. Furtado, leg., 1 male; Pará: *Kodormus* / *bruneosus* / Barber [handwritten] / Wygodzinsky det. [printed] ’64 [handwritten] // [printed label] Cachimbo E. [state of] Pará / Travassos-Oliveira / & Adão [leg.], 25/9-10-[1]956 // [framed printed label] CTIOC / N°. 847, 1 male; *Kodormus* / *bruneosus* / Barber [handwritten] / Wygodzinsky det. [printed] ’64 [handwritten] // [printed label] Cachimbo E. [state of] Pará / Travassos-Oliveira / & Adão [leg.], 25/9-10-[1]956 // [framed printed label] CTIOC / N°. 848, 1 male; *Kodormus* / *bruneosus* / Baber [handwritten] / Wygodzinsky det. [printed] ’64 [handwritten] // [printed label] Cachimbo E. [state of] Pará / Travassos-Oliveira / & Adão [leg.], 25/9-10-[1]956 // [printed label] Instituto Osvaldo Cruz // [handwritten label] desenhado [drawn] // [framed printed label] CTIOC / N°. 849, 1 male; *Kodormus* / *bruneosus* / Barber [handwritten] / Wygodzinsky det. [printed] ’64 [handwritten] // [handwritten label] Belém, Pará / M. Alvarenga / 1-1956 // [printed label] Instituto Osvaldo Cruz //[framed printed label] CTIOC / N°. 850, 1 male (CTIOC). Ecuador: Narupa, Napo Province, 1.200 m, 12.ii.1996, Juán Salvador leg., 1 male (MNRJ). French Guiana: Itoupé, DZ 570 m, 9.iii.2010, light trap, SEAG leg. 2 males and one female; N2, pk 79, 7.i.1996, PL, B. Hermier leg., 1 male; Degrad Corrèze, Route de régina, pk 62, 19.xii.1998, Kindl leg., 1 male; Degrad Kwata, iii.1995, PL, vesco JP leg., 1 male; Barrage petit Saut, 2.iii.1993, J–MB réc, 1 male; Grand Santi, PL, 29.iv.2000, P. Causse leg., 1 male; Laussat, PL, 11.ix.2010, light trap, SEAG leg., 1 female; D6, pk 37, 01.i.1998, light trap, B. Hermier leg., 1 female; Montagne des chevaux, PL, 22.xii.2008, light trap, SEAG leg., 1 female (J–MB). Peru: [red label with a smaller white label glued on it; both printed labels] Coll. R. I. Sc. N. B. [underlined by a black line] / Pérou [on the smaller white label] Peru 700 m / Chanchamayo / 20.X.1960 // [printed label] Kodormus / brunneus [*sic*] / Barber / JMaldonadoC.85 [1985] // *Kodormus* [printed] / *bruneosus* [handwritten] / Gil-Santana det. [printed] 19 [handwritten; 2019], 1 male (RBINS).

#### Diagnosis.

*Kodormusbruneosus* may be separated from most of the other species of the genus by the denticulate latero-distal angles of connexival segments II–VI and from *K.oscurus*, which although has a somewhat similar connexival structure, presents more prominent connexival latero-distal angles, and by their general coloration, which is generally brownish in *K.bruneosus* and darker in *K.oscurus*.

#### Description.

**Figures 55–62.**
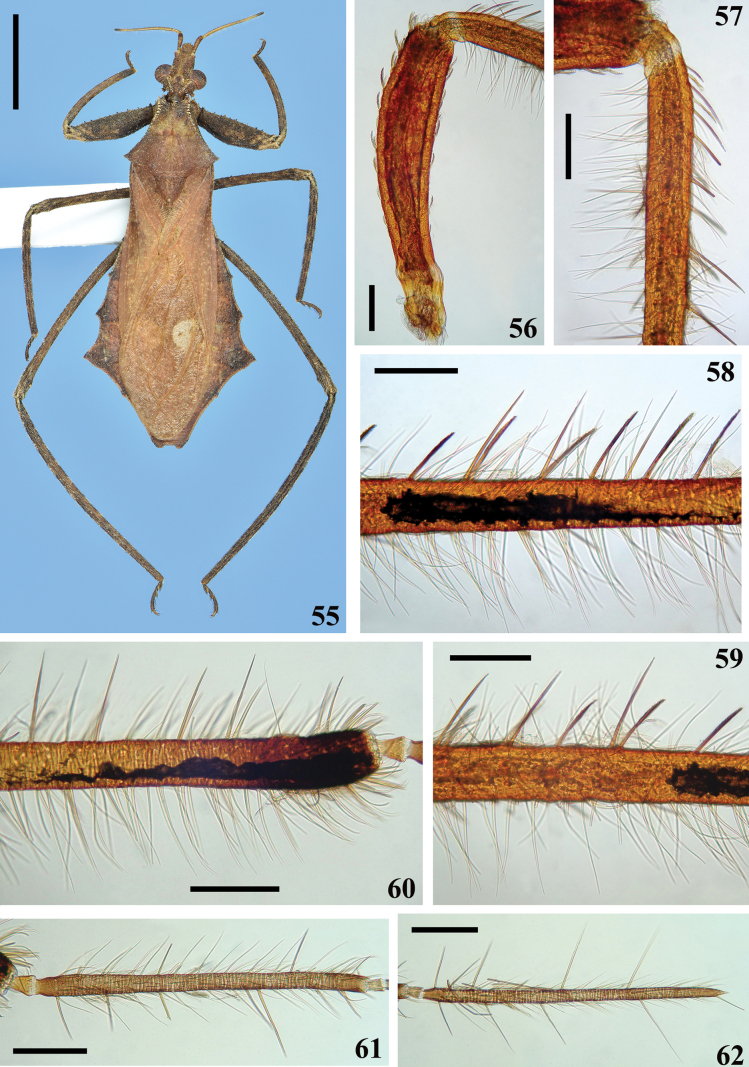
*Kodormusbruneosus*, male **55** specimen from Brazil, dorsal view **56–62** antennal segments or portions, lateral view **56** scape **57–60** pedicel **57** basal portion **58, 59** middle portion **59** somewhat distally **60** apical portion **61** basiflagellomere **62** distiflagellomere Scale bars: 5.0 mm (**55**); 0.2 mm (**56–62**).

**Figures 63–68.**
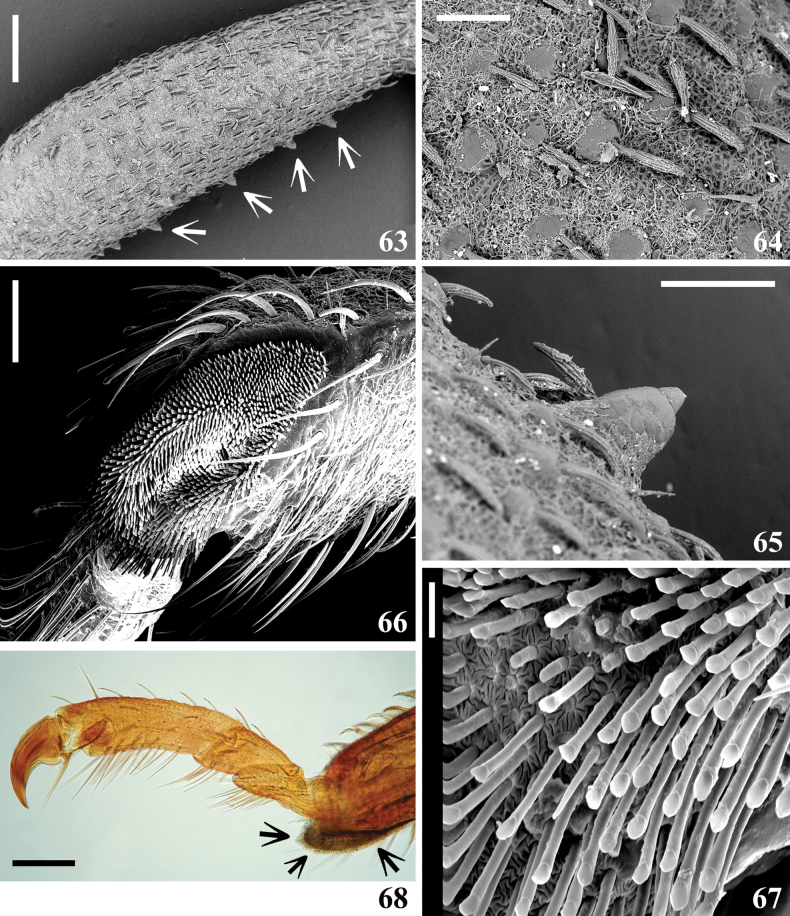
*Kodormusbruneosus*, male **63–65** fore femur, lateral view **63, 64** anterior surface **63** median portion, arrows point to ventral spiny rounded tubercles **64** portion of the integument **65** a spiny ventral rounded tubercle **66, 67** fore tibia, ventral view **66** apical pad **67** tenent hairs of a portion of the pad **68** apex of fore tibia and tarsus, lateral view, tibial pad pointed by arrows. Scale bars: 0.5 mm (**63**); 0.2 mm (**68**); 0.1 mm (**64–66**); 0.01 mm (**67**).

**Figures 69–74.**
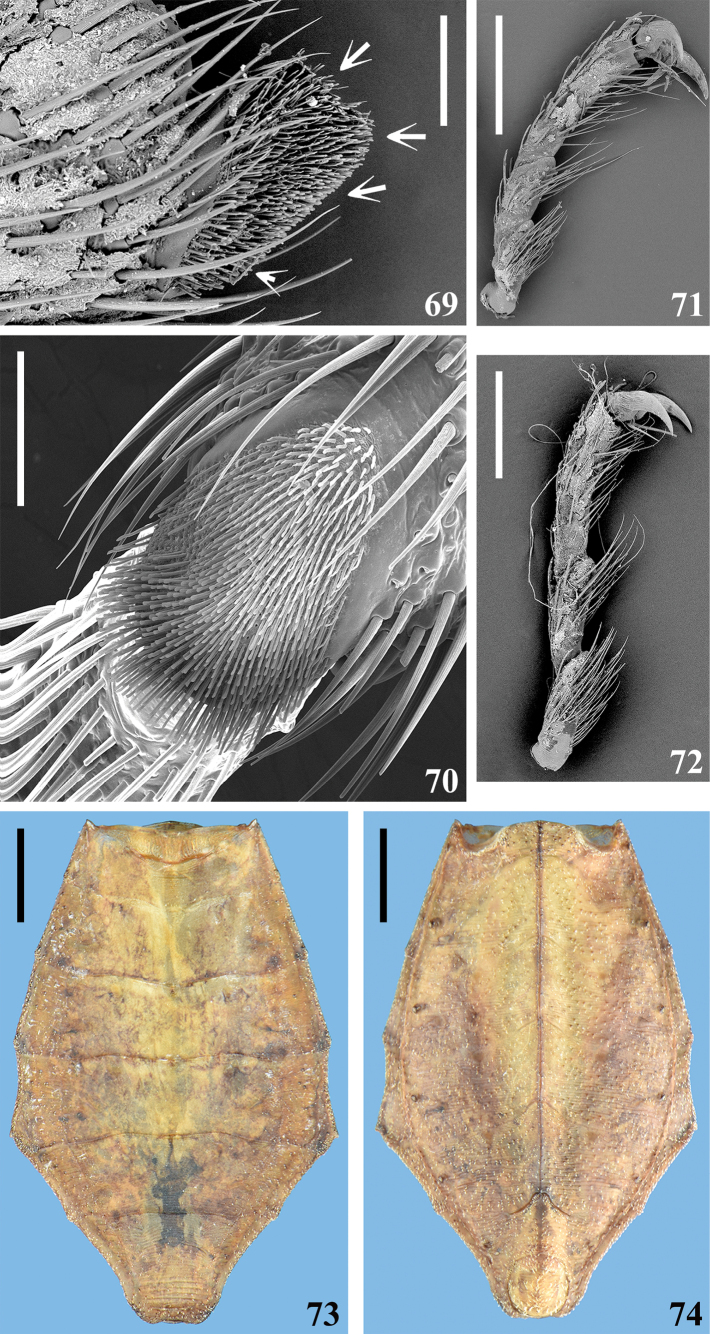
*Kodormusbruneosus*, male **69, 70** apex of middle tibia **69** lateral view, the arrows point to the distal pad **70** tibial pad, ventral view **71, 72** lateral view **71** middle tarsus **72** hind tarsus **73, 74** abdomen **73** dorsal view **74** ventral view. Scale bars: 2.0 mm (**73, 74**); 0.5 mm (**71, 72**); 0.1 mm (**69, 70**).

**Figures 75–85.**
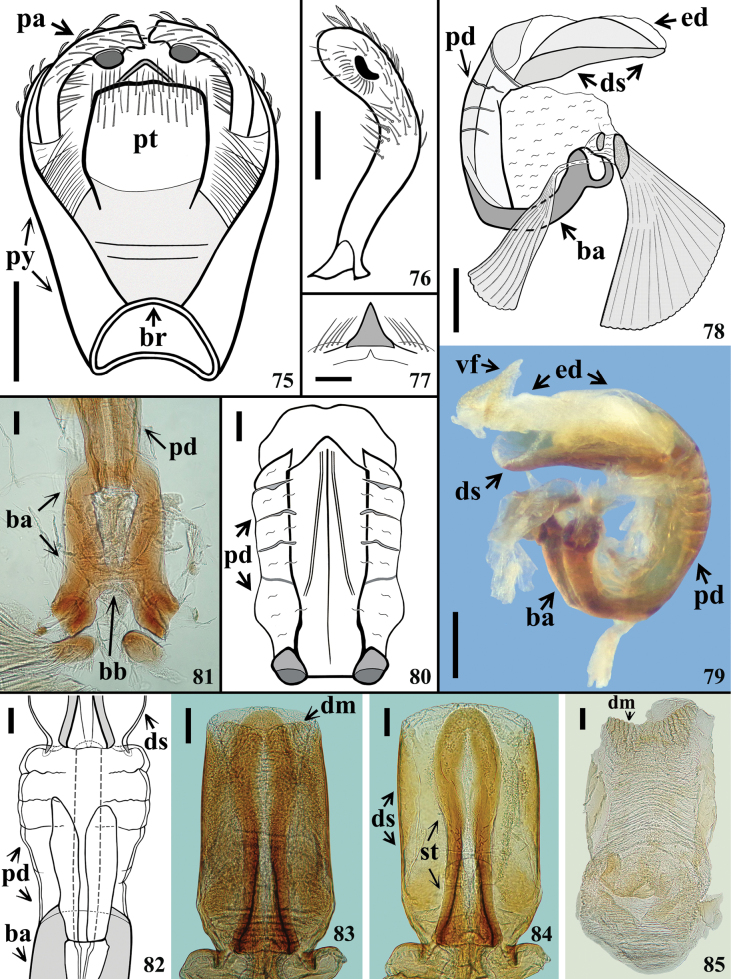
*Kodormusbruneosus*, male genitalia **75** genital capsule, dorsal view **76** left paramere, inner view **77** medial process of pygophore, anterior view **78–80** phallus **78, 79** lateral view **80** ventral view **81–85** dorsal view **81** articulatory apparatus and basal portion of pedicel **82** basal portion of basal plate arms, pedicel and basal portion of phallothecal sclerite and struts **83** dorsal phallothecal sclerite, struts and endosoma **84** dorsal phallothecal sclerite and struts (endosoma extracted) **85** endosoma. Abbreviations: **ba**: basal plate arm; **bb**: basal plate bridge; **br**: bridge; **dm**: distal margin of endosoma; **ds**: dorsal phallothecal sclerite; **ed**: endosoma; **pa**: paramere; **pd**: pedicel; **pt**: proctiger; **py**: pygophore; **st**: struts; **vf**: ventral fold of endosoma Scale bars: 0.5 mm (**75**); 0.3 mm (**76, 78, 79**); 0.2 mm (**77**); 0.1 mm (**80–85)**.

**Male** (Figs 51–85). Total length 17.5–21.5 mm; maximum width of abdomen (between apices of connexival prominences of segment V): 5.5–8.8 mm. ***Coloration*** (Figs 51, 53–55, 73, 74): generally brownish; in some individuals with some portions more darkened such as the fore lobe of pronotum, legs, prominences of connexivum, and ventral surface of abdomen. Antennal pedicel variably paler with apex darkened. Pale markings or portions variably scattered on head, apices of femora, basal portions of tibiae and sternites; the latter sometimes almost or completely paler. ***Structure*** and ***vestiture*** (Figs 51, 53–74): Postocular region of the head with two ramose setigerous processes posterolaterally on each side, very close to each other, the most posterior one slightly above of the other. Setigerous tubercles on serial line of postocular region of head, anterior collar and single rows on the lateral margins of fore lobe of pronotum variable in size and coloration among individuals, larger and pale to whitish or smaller and darker. Tubercles on disc of fore lobe flat, rounded. Humeral angle short, spiniform (Figs 51, 53–55). Process of scutellum short. Membrane of hemelytra varying from not reaching to slightly surpassing apex of abdomen (Figs 51, 53–55). Fore tarsus three-segmented (Fig. 68). Lateroapical margins of connexivum more or less prominent among individuals; that on segment V is sometimes apically curved downward (Figs 51, 53–55, 73, 74). ***Male genitalia*** (Figs 74–85): medial process of pygophore enlarged; triangular in anterior view (Fig. 77).

**Figures 86–90.**
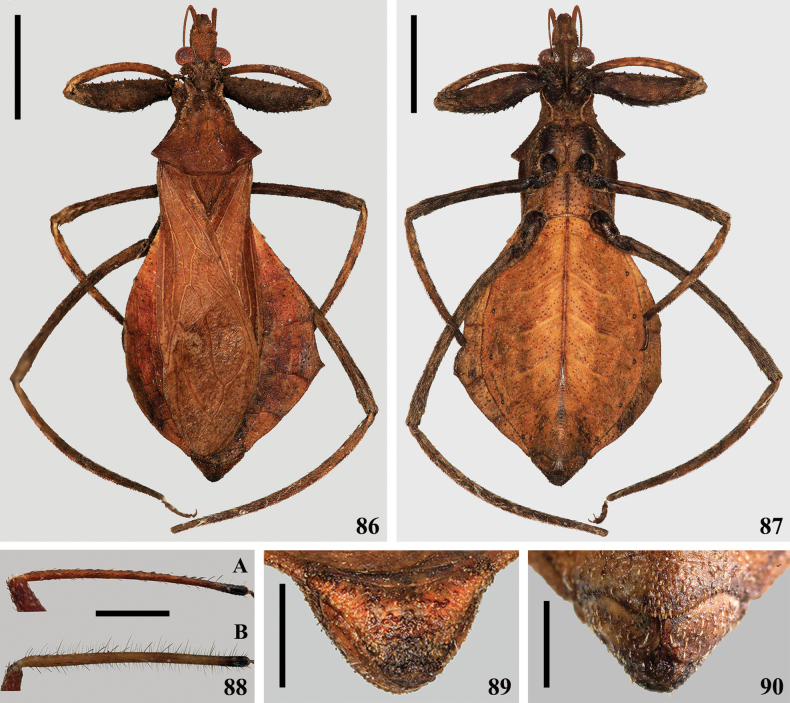
*Kodormusbruneosus***86, 87** female **86** dorsal view **87** ventral view **88** antennal pedicels of a female (**A**) and of a male (**B**), lateral views **89, 90** female genitalia, external view **89** posterior view **90** ventral view. Scale bars: 5.0 mm (**86, 87**); 1.0 mm (**88**); 0.5 mm (**89, 90**).

**Female** (Figs 86–90): Total length: 22–23 mm; maximum width of abdomen between apices of connexival prominences of segment V: 9–11 mm. Similar to male in general (Figs 86, 87). Antennal pedicel with scattered very short and sparse scale-like setae, and a few thin, long setae distally (Fig. 88, A). Abdomen very wide, with a maximum width on segment V (Figs 86, 87). Membrane of hemelytra not reaching apex of abdomen (Fig. 86); genital area visible from above, cone shaped and acute (Fig. 86). ***Female genitalia***: external genitalia as in Figs 89, 90.

#### Comments.

Barber (1930) recorded the tibial pad as absent at the apex of fore tibia in *K.bruneosus*. Giacchi (1985), when redescribed the male of this species, did not mention the presence or absence of pads on the tibiae. However, we have recorded the presence of tibial pads at apices of fore and middle tibiae in all specimens of *K.bruneosus* studied here (Figs 66–70). Our observation is in accordance with Weirauch (2007) who also recorded tibial pads (as fossula spongiosa) as present both in fore and middle tibiae of *K.bruneosus*.

The description of *K.bruneosus* by Barber (1930) seems to have been based only on the male type (s), because no detail was given concerning the female cited as “Paratype”. There was no mention about differences between sexes and neither about the genital portions. While Barber (1930) recorded the pedicel as densely setose, as seen in males (Figs 57–60), he did not mention that, accordingly with the females examined here, it is remarkably less setose in the females (Fig. 88, A). Additionally, the females were generally larger, with wider abdomens (Figs 86, 87).

When recording *K.bruneosus* from Colombia, Forero (2006) listed Brazil as a country of occurrence of the species too. However, this supposed record was based on Wygodzinsky and Giacchi (1994), who actually recorded only *Kodormus* from Brazil, not specifying any species of the genus. Their record may possibly have been based on *K.barberi*, the only species recorded from Brazil so far. This assertion was confirmed to the first author (HRG-S) by D. Forero (pers. inform.). Therefore, the first proven record of *K.bruneosus* from this country is provided here.

#### Distribution.

Panama, Guyana (Barber 1930), French Guiana (Villiers 1971), Trinidad and Tobago, Venezuela (Giacchi 1985), Bolivia (Maldonado 1990), Colombia (Forero 2004, 2006), Ecuador and Peru (Gil-Santana and Husemann 2023).

#### New record.

Brazil (States of Maranhão, Mato Grosso and Pará).

### ﻿ Kodormus davidmartinsi sp. nov.

Taxon classificationAnimaliaHemipteraReduviidae

9DEEB7E4-18ED-57D8-9733-4331BADC5D62

https://zoobank.org/0EB02FCF-1149-40D3-B7F6-5451A1588C4B

Figs 91–95 , 96–104

Kodormus barberi ; Gil-Santana and Alencar 2001: 173 [checklist; misidentification].

#### Notes.

Gil-Santana and Alencar (2001) based on a male specimen from a Natural Reserve in Linhares, Espírito Santo State, Brazil, included *Kodormusbarberi* in a checklist of Reduviidae of this locality. However, a re-examination of the specimen from Linhares made it clear that it belongs to the new species, *K.davidmartinsi* sp. nov., with the designation of this specimen as the holotype. An additional specimen from the same locality was included as a paratype.

#### Type material examined.

Brazil, Espírito Santo: Linhares, Reserva Natural Vale, 19°09'S, 40°04'W, José Simplício dos Santos leg., ***male holotype***, xi.1990 (MNRJ); same locality and collector, 1 ***male paratype***, 11.xii.1987, CTIOC n° 13832 (CTIOC).

#### Diagnosis.

*Kodormusdavidmartinsi* sp. nov. and *K.barberi* may be separated from other species of the genus by the presence of connexival margins of segments III–V lobulated. These species may be separated from each other by the larger lobulated portion of connexival segment V in *K.davidmartinsi* sp. nov. Additionally, *K.davidmartinsi* sp. nov. has smaller integumental setigerous spiniferous processes, shorter processes of disc of fore lobe of pronotum, humeral angles, scutellum and rounded latero-distal margins of abdominal segment VII. In male genitalia, the medial process of pygophore in anterior view, is subtriangular in *K.davidmartinsi* sp. nov. and spiniform in *K.barberi*.

#### Description.

**Figures 91–95.**
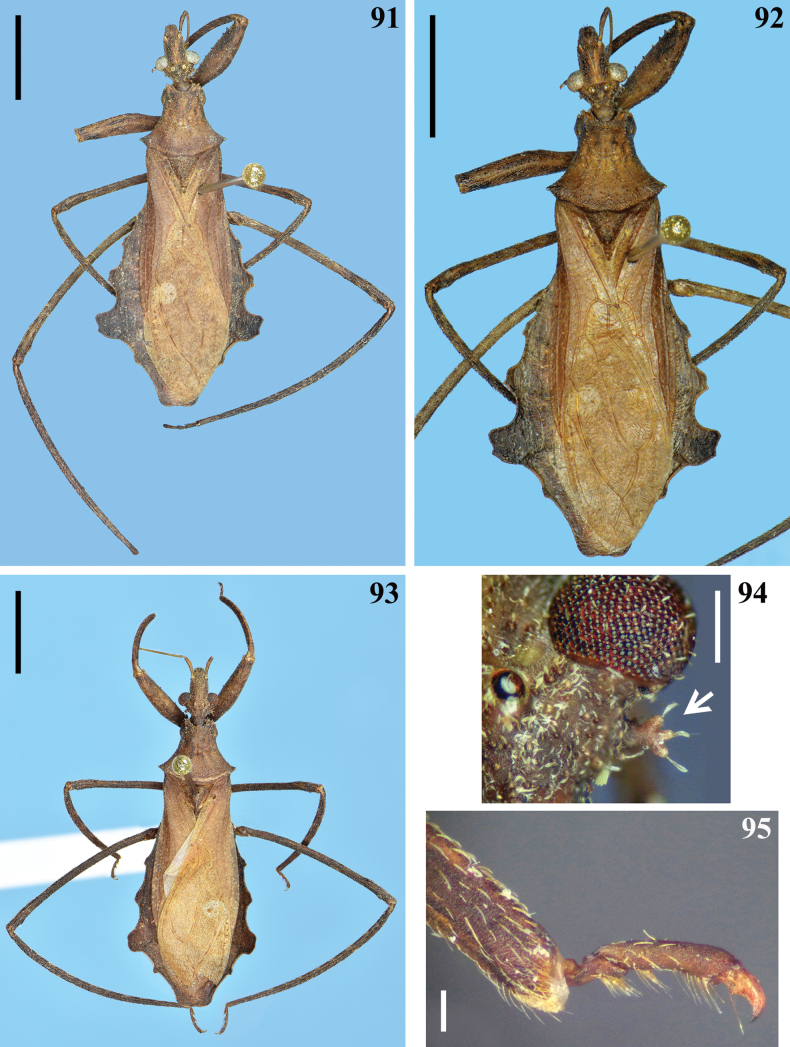
*Kodormusdavidmartinsi* sp. nov., male **91–94** dorsal view **91, 92** holotype **93** paratype **94** postocular portion of the head, the arrow points to a posterolateral ramose setigerous process **95** fore leg, apex of tibia and tarsus, lateral view. Scale bars: 5.0 mm (**91–93**); 0.5 mm (**94**); 0.2 mm (**95**).

**Figures 96–104.**
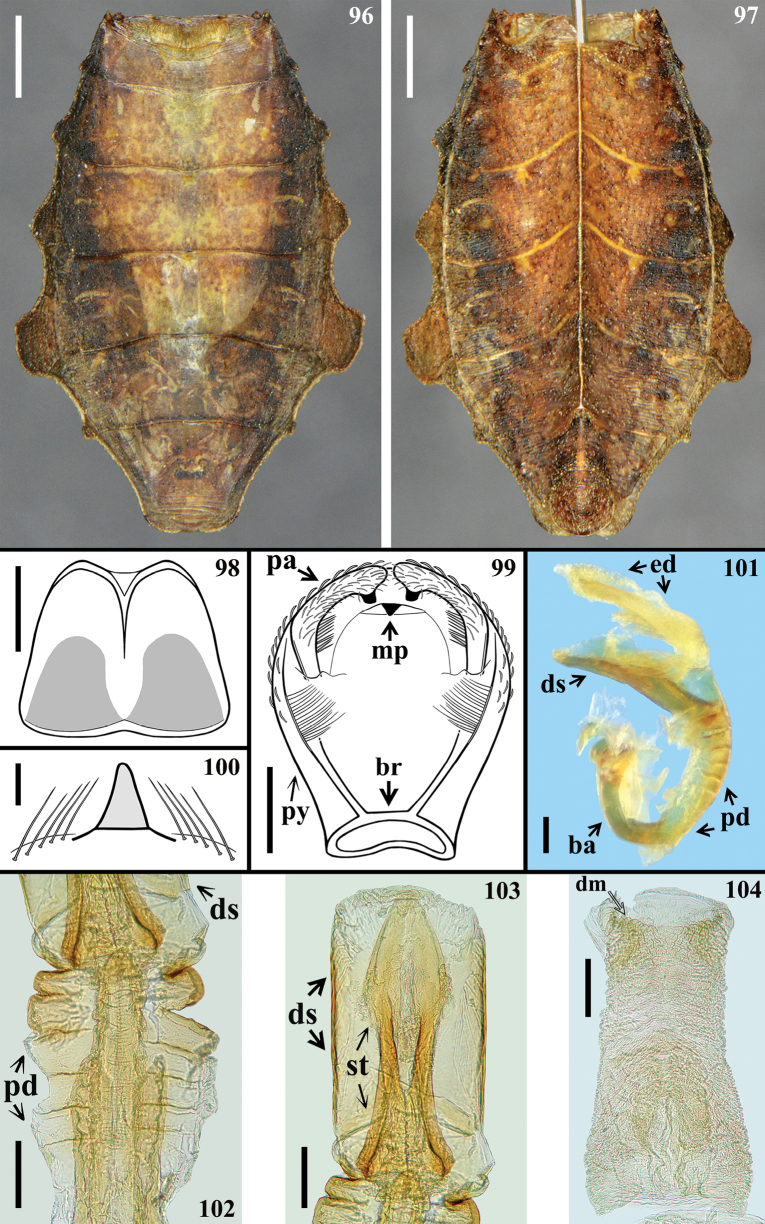
*Kodormusdavidmartinsi* sp. nov., male **96, 97** abdomen of the paratype **96** dorsal view **97, 98** ventral view **98** eighth sternite **99–104** male genitalia **99** pygophore and parameres (proctiger and phallus extracted), dorsal view **100** medial process of pygophore, anterior view **101** phallus, lateral view **102–104** dorsal view **102** pedicel and basal portion of phallothecal sclerite and struts **103** dorsal phallothecal sclerite and struts (endosoma extracted) **104** endosoma. Abbreviations: **ba**: basal plate arm; **br**: bridge; **dm**: distal margin of endosoma; **ds**: dorsal phallothecal sclerite; **ed**: endosoma; **pa**: paramere; **pd**: pedicel; **mp**: medial process of pygophore; **py**: pygophore; **st**: struts. Scale bars: 2.0 mm (**96, 97**); 0.5 mm (**98, 99**); 0.2 mm (**101–104**); 0.1 mm (**100**).

**Male.** Figs 91–104. Measurements (mm) (holotype / paratype): ***Total length***: 20.5 / 21.5; ***head***: total length (excluding neck, lateral view): 3.2 / 3.1; maximum width across eyes: 2.4 / 2.7; length of anteocular portion: 1.6 / 1.6; length of postocular portion: 0.8 / 0.7; interocular space (synthlipsis): 1.0 / 1.1; transverse width of right eye: 0.7 / 0.8; length of right eye: 0.8 / 0.9; lengths of antennal segments: scape: 1.3 / 1.4; pedicel: 3.0 / 3.0; basiflagellomere 0.8 / 0.6; distiflagellomere: 0.8 [approx.; very curved] / absent; lengths of labial segments: II [first visible]: 1.6 / 1.7; III: 1.4 / 1.6; IV: 0.9 / 0.9. ***Thorax***: pronotum: length of fore lobe (at midline): 1.9 / 1.9; length of hind lobe (at midline): 1.8 / 1.8; width at posterior margin: 4.7 / 5.0. Fore legs: length of femur: 5.4 / 5.6; maximum width of femur at mid portion: 1.3 / 1.4; length of tibia: 5.2 / 5.4; length of pad: 0.1 / 0.2; length of tarsus: 0.7 / 0.8; middle legs: length of femur: 6.5 / 7.2; maximum width of femur at mid portion: 0.45 / 0.45; length of tibia: 6.4 / 7.5; length of pad: 0.2 / 0.2; length of tarsus: 1.5 / 1.5; hind legs: length of femur: 10.0 / 11.5; maximum width of femur at mid portion: 0.5 / 0.5; length of tibia: 12.8 / 13.8; length of tarsus: 1.8 / 1.9. ***Abdomen***: length: 11.2 / 11.7; maximum width (measured between outer margins of anterior portion of sternite V): 6.1 / 6.5; maximum distance between outer margins of lobulated prominence of connexival segment V: 9.2 / 8.8. ***Coloration*** (Figs 91–93, 96, 97): generally dark brownish with ill-defined scattered pale portions or markings on head, femora and sternites; hemelytra, except basal portion, paler, more in the paratype. Antenna pale brownish; scape with irregular scattered dark small markings; apex of pedicel dark. The following portions variably paler: glabrous areas of head and fore femora, more intensively in the holotype; inferior margin of fore supracoxal lobe; upper portions of lateral surfaces and apices of femora; a pair of linear markings at basal portions of tibiae; median portion of tergites I–V (Fig. 96), progressively in less extent from the former to the latter; margins of sternites between segments II–VI; on the latter, at each lateral side, a pair of rounded (paratype) or irregular (holotype) small markings on basal margin of these segments and another similar markings between basal and distal margins (Fig. 97). ***Structure*** and ***vestiture*** (Figs 91–97): Postocular region of the head with only one ramose setigerous process posterolaterally at each side (Fig. 94). Processes of scutellum and humeri short. Fore trochanters with two pairs of spiny tubercles on internal surface. Fore femora with a basal group from two to four spiny, relatively small, rounded tubercles, a midline row with eight spiny rounded tubercles and two (holotype) and four (paratype) others close to this row on anterior surface. Fore tarsi bi-segmented; the second segment ~ 3× as long as the first segment (Fig. 95). Connexival margins of segments III–VI lobulated; those on segments III–V have the external margin sinuated at median portion and a short spiny prominence at latero-distal angle in holotype, while in paratype the external margin is faintly curved without spiny prominences (Figs 91–93, 96, 97). Membrane of hemelytra not reaching apex of abdomen (Figs 91–93). Lateroapical margins of abdomen curved, slightly prominent (Fig. 96). ***Male terminalia*** (Figs 98–104): medial process of pygophore subtriangular in anterior view (Fig. 100).

#### Etymology.

The new species is named in honor of Dr. David dos Santos Martins, researcher of the Instituto Capixaba de Pesquisa, Assistência Técnica e Extensão Rural (INCAPER), Vitória, Espírito Santo, Brazil, for his great contribution to the knowledge of the entomofauna of the State of Espírito Santo where the new species was found.

#### Distribution.

Brazil (State of Espírito Santo).

### ﻿ Kodormus oscurus

Taxon classificationAnimaliaHemipteraReduviidae

Maldonado & Bérenger, 1996

1AD542AC-D9A3-5B16-981B-B68879476D71

Figs 105–110

Kodormus oscurus Maldonado & Berénger, 1996 in Bérenger and Maldonado 1996: 35–37, figs 1–7 [description]; Forero 2004: 166 [citation].

#### Notes.

*Kodormusoscurus* was described based on 10 males collected in Bolivia (Bérenger and Maldonado 1996).

#### Type material examined.

Bolivia: Ixiamas, Beni, oct. 1993, 270 m, Bleuzen leg., 1 ***male paratype***; Pucara, Beni, oct. 1993, 750 m, Bleuzen leg., 1 ***male paratype*** (J-MB).

#### Diagnosis.

*Kodormusoscurus* is separated by its general coloration, which is generally dark brown, while in the other species of *Kodormus*, it is generally brownish. *Kodormusoscurus* seems closer to *K.bruneosus*, based on the denticulate latero-distal angles of connexival segments II–VI, but in the latter species these angles are less prominent.

#### Description.

**Figures 105–110.**
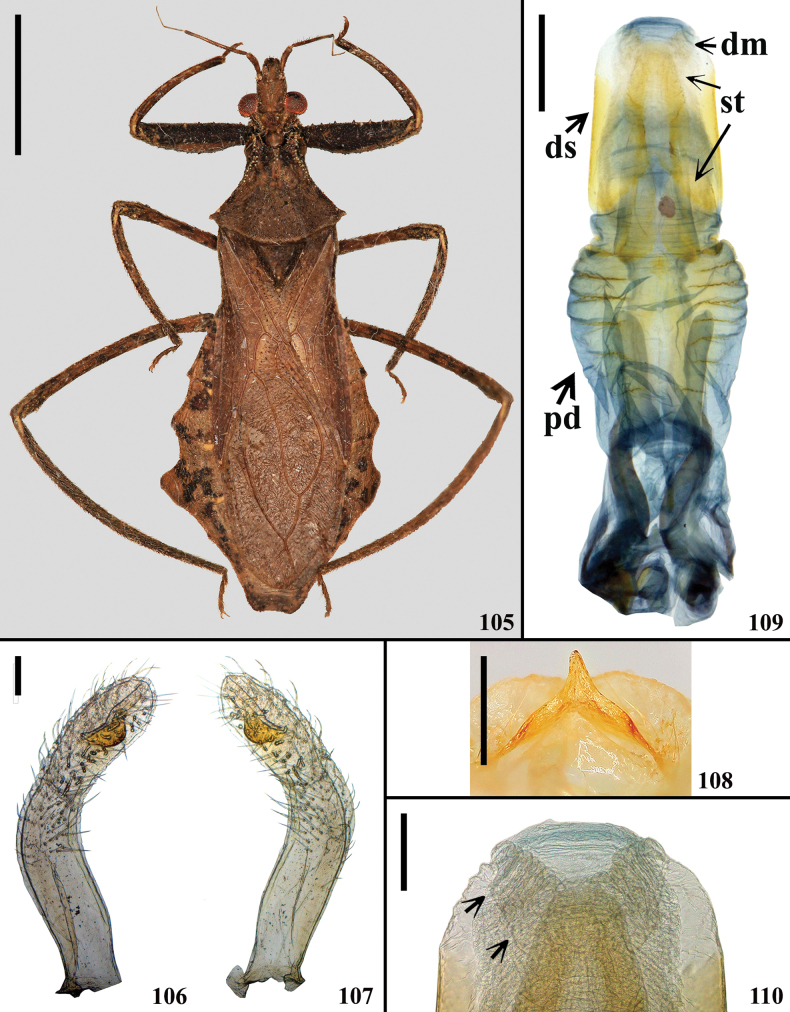
*Kodormusoscurus* Maldonado & Bérenger, 1996, male paratype **105–107** dorsal view **106–110** male genitalia **106, 107** parameres **106** right **107** left **108** medial process of pygophore, anterodorsal view **109, 110** dorsal view **109** phallus **110** apex of dorsal phallothecal sclerite, struts and endosoma, arrows point to the distal margin of endosoma. Abbreviations: **dm**: distal margin of endosoma; **ds**: dorsal phallothecal sclerite; **pd**: pedicel; **st**: struts. Scale bars: 5.0 mm (**105**); 0.5 mm (**108, 109**); 0.2 mm (**106, 107**); 0.1 mm (**110**).

**Male.** Figs 105–110. ***Total length***: 19.5–25 mm. ***Coloration*** (Fig. 105): Generally dark brownish; hemelytra and sternites reddish brown, slightly paler than dark brownish portions. ***Structure*** and ***vestiture*** (Fig. 105): Postocular region of the head with two ramose setigerous processes posterolaterally at each side, very close to each other, the most posterior one slightly above of the other. Setigerous tubercles on serial line of postocular region of head very numerous, pale and conspicuous; those on the lateral margins of fore lobe of pronotum forming an irregular row, most of them pale. Tubercles on disc of fore lobe flat, rounded. Humeral angle short, spiniform. Process of scutellum short. Membrane of hemelytra varying from not reaching to reaching apex of abdomen. Fore tarsus three-segmented. Lateroapical margins of connexivum conspicuously prominent; those on segments IV and V are apically curved downward in some individuals. ***Male genitalia*** (Figs 106–110): medial process of pygophore enlarged; triangular in anterior view (Fig. 108).

#### Distribution.

Bolivia (Bérenger and Maldonado 1996).

## ﻿Discussion

Because most species of Stenopodainae are known from light trap collecting, with males being captured much more commonly than females (Giacchi 1987; Bérenger 2001; Schuh and Weirauch 2020), the female of many species remains unknown. In *Kodormus*, only females of *K.bruneosus* were collected. Barber (1930) included a female “Paratype” in his description of *K.bruneosus*, but did not provide any specific comments about it, such as differences in size or in the antennal vestiture, as it commonly occurs among reduviids (Giacchi 1987). In this study, females of *K.bruneosus* from French Guiana were examined by the second author and shown to be generally larger, with wider abdomens. Additionally, the antennal vestiture of the pedicel was quite diverse, much more differentiated in the males, as described above. Nevertheless, there is a need to obtain females of the other species to a better knowledge of range of variation or sexual dimorphism between males and females of species of *Kodormus*.

Barber (1930) in his description of *Kodormus* recorded the pad as absent at the apex of fore tibia. Although the presence or absence of a pad on the fore and/or middle tibiae is an important feature for separating the genera of Stenopodainae, there is no further mention about the presence or absence of the pad on tibiae of *Kodormus* in the redescription of this genus by Giacchi (1985), and in diagnosis and/or keys in which it was included (Costa Lima and Campos Seabra 1944; Wygodzinsky and Giacchi 1994; Forero 2004; Gil-Santana et al. 2015; Gil-Santana and Oliveira 2016). Only in the morphological comparative study of Weirauch (2007: 159) it was recorded on fore and middle tibiae of *K.bruneosus*. However, we confirmed the presence of the pad on fore and middle tibiae in all species of *Kodormus* studied here (e.g., Figs 31, 66, 68–70). Therefore, this feature was included in accordance with our observations in the description of the genus presented above. On the other hand, the fore tarsi are bi-segmented in *K.barberi* and *K.davidmartinsi* sp. nov. (Figs 32, 33, 95) while in *K.bruneosus* (Fig. 68) and *K.oscurus* it is tri-segmented, as usual in reduviids. Among New World Stenopodainae, the bi-segmented fore tarsi are included among the diagnostic characteristics of *Rhyparoclopius* Stål, 1868 (Forero 2004; Gil-Santana 2012; Gil-Santana et al. 2015; Gil-Santana and Oliveira 2016). Interestingly, when describing *Kodormus*, Barber (1930) stated that this genus had no close affinity to any other genus of Stenopodainae, but seemed close to *Rhyparoclopius* in the broader character of the body, while Gil-Santana and Oliveira (2016) highlighted that these two genera were among those which have the fore femora moderately to strongly incrassate (at least twice as thick as the mid and hind femora), and together with *Otiodactylus* Pinto, 1927, all three have the antennal scape shorter than the length of the anteocular portion of the head. Bérenger and Maldonado (1996) considered that among Neotropical genera with enlargement of the connexival margins, such as *Rhyparoclopius* and *Otiodactylus*, the latter seemed to be the closest to *Kodormus*. Nevertheless, the significance of these similiarities needs a more thorough evaluation, including cladistic studies in order to clarify the systematic relationship among the genera of Stenopodainae.

Several works, mostly authored by J.C. Giacchi, summarized by Gil-Santana et al. (2015), have described the male genitalia of many species of American Stenopodainae. Differences between or among species in the same genus, involving mostly the parameres, the shape of the dorsal phallothecal plate, struts and the processes or sclerotizations of the endosoma, have been recorded (e.g., Giacchi 1969; Gil-Santana 2012). However, in the two species of *Nitornus* Stål, 1859, which are quite diverse in several morphological features, the male genitalia of both was very similar (Gil-Santana 2016). Among the species of *Kodormus* examined here, the structure of the male genitalia, including the pygophore, parameres and phallus, was also very similar. The medial process of pygophore was the only structure which was recorded as being slightly different in shape (in its anterior view), more prominently in *K.barberi*. Thus, it seems that the male genitalia of *Kodormus* has limited taxonomic utility.

## Supplementary Material

XML Treatment for Kodormus

XML Treatment for Kodormus barberi

XML Treatment for Kodormus bruneosus

XML Treatment for Kodormus davidmartinsi

XML Treatment for Kodormus oscurus

## ﻿Additional information

### Author ORCIDs

Hélcio R. Gil-Santana https://orcid.org/0000-0002-0544-348X

Jean-Michel Bérenger https://orcid.org/0000-0002-8497-2792

Jader Oliveira https://orcid.org/0000-0002-2588-1911

### Data availability

All of the data that support the findings of this study are available in the main text.
